# Health Educator Participation in Virtual Micro-Credentialing Increases Physical Activity in Public Health Competencies

**DOI:** 10.3389/fpubh.2021.780618

**Published:** 2021-12-07

**Authors:** Anna Dysart, Laura E. Balis, Bryce T. Daniels, Samantha M. Harden

**Affiliations:** ^1^Department of Human Nutrition, Foods, and Exercise, Virginia Tech, Blacksburg, VA, United States; ^2^Louisville Center, Pacific Institute for Research and Evaluation, Louisville, KY, United States; ^3^Health, Human Performance, and Recreation, University of Arkansas, Fayetteville, AR, United States

**Keywords:** physical activity, public health, competency-based trainings, health educators, Cooperative Extension

## Abstract

**Background:** Physical activity is an important component of leading a healthy life. Public health is one of the nine major sectors for disseminating information about physical activity and increasing the physical activity of the general public.

**Purpose:** Increase competency among Cooperative Extension agents (i.e., public health workers) on selecting, delivering, and evaluating physical activity programs through a theory-based online training program.

**Methods:** Cooperative Extension agents from two states were invited to participate via statewide listservs. Participants were invited to attend sessions, complete competency checks, and between-session assignments each week. The study was conducted using a video conferencing platform. The intervention was 9 weeks from June to July 2020 and had 130 participants. Pre- and post-program surveys included physical activity competencies and validated scales for flourishing and physical activity status. Data for competencies pre and post were analyzed using the Wilcoxon signed rank test, *p* < 0.01. Physical activity and flourishing pre and post were compared using *t*-tests, *p* < 0.05.

**Results:** Physical activity in public health competency increased significantly (*p* < 0.00) as did agents' personal physical activity levels (*p* < 0.05). Changes in flourishing were not significant (*p* < 0.09) but trended in the hypothesized direction.

**Conclusions:** The online competency-based training program significantly improved Cooperative Extension agents' knowledge of physical activity guidelines and physical activity program implementation. Future work is needed related to the scalability of the training program.

## Introduction

Physical activity is a recognized way to stay healthier and reduce comorbidities for people of all ages and abilities (1–4). The scientific evidence and best practices of the Physical Activity Guidelines for Americans (PAGA) have been translated for public consumption via the United States' government-sponsored Move Your Way campaign (2). Despite these dissemination efforts and benefits, only 26% of American men and 19% of American women meet the recommendations for physical activity (4). The public health sector is one of the nine major disseminating sectors of physical activity messaging and programming, with others including faith-based organizations and health care, as discussed in the National Physical Activity Plan (4, 5). Public health workers and officials can help promote the increase of physical activity throughout the community, as well as assist in tracking the proportion of the population that is physically active (3, 4).

One public health sector that promotes healthy lifestyles is the national Cooperative Extension (herein: extension) system (6–8). As part of the historic land-grant university system existing within all states and territories within the United States, the system is federally funded and reaches millions of Americans each year (8). County-based extension professionals (herein: agents) have a unique opportunity to engage with communities improve individual and community health, safety, and food production (8).

The job responsibilities of agents, like most public health workers, continuously change to match public health needs (9). Notably, extension has strong roots in rural settings (8), as it emerged from farming practices, and only recently began to translate physical activity messaging and interventions within their programming (10–12). This necessitates continued education structured around the knowledge they need to do their job (i.e., competencies) (13–15). Travel budgets, timing, and other constraints have made an online format a more appealing option for continuing education in recent times (14, 16). The COVID-19 pandemic further highlighted the need for virtual training protocols for wide-reaching systems.

Previous research indicates that online trainings have myriad benefits (e.g., reaching a broad geographic region, satisfaction with content) and challenges (e.g., sufficient internet access and cost) (14, 16, 17). Notably, asynchronous trainings have been further critiqued for the lack of peer-to-peer interaction and interaction with the training platform (i.e., quizzes, role-playing, etc.) (16). Research is lacking in synchronous online trainings for public health workers, though there has been some research showing that synchronous online trainings can be a beneficial learning tool for teachers (18) and other health professionals (17). Trainings that allow participants to have multiple exposures to topics and are more interactive, including practicing skills through homework assignments, result in higher impacts on outcomes (19–21).

Explorations of the structure of trainings show that competency-based trainings result in significant improvement in the competency domains (13) and build confidence (15) with public health workers (13, 15). Having a competency-based training that included programming suited for adult learners and participant interaction was found to be helpful in a community–academic initiative training for community health workers to demonstrate the larger context of their work (15). This also increased their desire to take what they learned and implement it with their own participants (15).

Public health workers who participated in trainings that focused on both core (knowledge) competencies and skill-based leadership competencies were found to self-report significantly improved competency status (13). Improved competency status has also been correlated to experience and frequency of use of the knowledge obtained through the trainings (13, 22). However, trainings for public health workers need to account for varying trainee characteristics (13, 15, 22). For example, those newer to the field are more likely to benefit from training (13, 22); those who are more physically active themselves are more likely to deliver physical activity programming (11); and those who are flourishing (strong overall sense of well-being and goodness in all sectors of a person's life), also have higher job satisfaction and work engagement (23). Flourishing and physical activity are intertwined as well, as physical and mental health is one of the domains of flourishing (24) and physical activity promotes physical and mental health (2). Many fields, including medicine, have adopted micro-credentialing as a form of professional development or continuing education that demonstrates a valuable skill (25).

Taken together, Cooperative Extension agents have the potential to promote physical activity if they are trained on doing so; the training needs to be synchronous and dynamic to improve personal and professional behaviors. While eventual physical activity program uptake by agents is the downstream goal of this work, the purpose of this study was to test the initial reach, and efficacy of a micro-credentialing program developed and evaluated during the 2020 pandemic to increase the competence of extension agents in physical activity guidelines and programming. Secondary aims included improving extension agents' perceptions of flourishing (overall sense of well-being) and their own physical activity levels since these are predictors of job satisfaction and program adoption, respectively.

## Methods

### Recruitment and Participants

This was a multi-state collaboration in which participants were recruited from Virginia and Arkansas state systems through extension listserv emails detailing the program goals, objectives, and session times. The training was held during working hours, but was not tied directly to job expectations or performance reviews. While agents were the main target of the program, other extension employees were not excluded from participating. There are five key roles in the extension system, administrators are individuals who oversee extension staff and budget (8) (and usually have a role in agent annual reviews); program leaders are individuals who oversee program teams (e.g., issue-dependent groups such as Food, Nutrition, and Health); specialists are university-based (8) and typically have a terminal doctoral degree; agents are county-based and responsible for responding to community needs (11); volunteers undergo program specific training provided by their agent; and “other” personnel included area coordinators and support staff.

Reach was operationalized as the total proportion of eligible participants that joined, ongoing reach of training materials (i.e., attendance and completion of the program), and the representativeness of the participants (26). This study was reviewed by the University Institutional Review Board (IRB) and determined to be exempt from IRB review as it did not meet the criteria to be considered human subjects research (i.e., federal exemption for normal activities within educational setting).

### Micro-Credentialing Program

The 9-week micro-credentialing program was titled Physical Activity in Cooperative Extension (PACE)—using the tagline, *Let's set the PACE!* Weekly synchronous sessions were approximately 60 min and held on the Zoom web conferencing platform. This platform has been used for other training within each state system prior to this micro-credentialing program. All sessions, assessments, and evaluation were Internet-based.

Evidence-informed components of the curricula include learner-centered approaches (19–21), educational theories (27), and group dynamics (28) (see Table 1). Between sessions, attendees were asked to complete asynchronous activities for mastery experiences related to each session topic. For discussion and application, asynchronous activities were discussed further in breakout rooms during the next Zoom session, or embedded within the between session assignments. These assignments were used as teach-back (27) moments generated in alignment with each predetermined objective (see Table 1). Post-session homework assignments were assessed for completion, not graded for accuracy (and therefore not an outcome measure of this trial but rather a feature of the micro-credentialing program). Two examples of these between-session tasks are asking participants to identify if their state extension strategic plan includes physical activity and to view the Move Your Way PAG campaign materials. The goal was to use these assignments to reinforcement ideas discussed each week. For the audit and feedback portion of each session, results of these assignments were discussed at the beginning of the next session, with the right answers being discussed (where applicable) as well as some of the answers to the open-ended questions.

**Table 1:** Outline for physical activity in Cooperative Extension: Let's set the PACE!.

**9 h direct contact; 9 h outside training**			
**Session title**	**Competencies**	**Breakout**	**Non-contact hours**
	By the end of this session, participants will be able to:		
Introduction to PACE/physical activity recommendations and types	1. Knowledge of training opportunities available in extension. 2. Ability to understand how specific training and technical assistance will help agents set the PACE.	• Active name game • Why did you join? • Come back to large group and share • Create a team name! • Exchange email addresses	• Visit move your way campaign site • Pick a target audience (e.g., youth, adults, older adults) and determine what resources are available for you to guide them regarding the PAG • Complete competency check
	1. Understand physical activity recommendations (2, 29) 2. Describe how PAG were developed 3. Knowledge of and ability to describe the four domains of physical activity: activities of daily living, active transportation, recreation or leisure activities, and occupational activities 4. Defend the importance of physical activity for their participants 5. Ability to select or modify physical activity programs that are appropriate to meet the needs of a specific community or population (29)	• Share how/if you are meeting PAG • What do you think will work in your community for PAGA • How are you already promoting PACE?	
Extension's role in physical activity promotion	1. Describe the framework for health and wellness 2. Relate the delivery of physical activity to the overall mission of Cooperative Extension 3. Define scope of work (educational and experiential not personal trainer or physical therapist), liability within extensions	• What was your favorite way to move as a kid? • How has that changed to now? • What is your perception that PA is a shared value in your state (other educators, admin, etc.)?	• Review your state extension strategic plan. Does it include physical activity? If so, what does it say? • Take competency check
Selecting and adapting evidence-based physical activity programs	1. Levels of evidence, what works? (29) 2. How do we choose programs? (29) 3. Adaptation vs. deviation	• Share an example of one deviation and one adaptation you have done over the years	• Search one of the provided repositories for an evidence-based program you might use in your community. What is its level of evidence? • Take competency check
Behavior change strategies	1. Knowledge of at least two behavioral strategies, such as goal setting or self-monitoring to be considered in planning PA interventions (29) 2. Understand the impact of behavior change strategies (29, 30) 3. Select and differentiate appropriate behavior change strategies (29, 30) 4. Assess if behavior change strategies are incorporated within programming 5. Describe group dynamics constructs 6. Apply group dynamic based-principles within existing Cooperative Extension programming	• When have you seen group dynamics in action? • Which of the discussed strategies have you used before? • What seems new and exciting to implement in your programming?	• Think of a program you offer to a “group” of people, but haven't facilitated group dynamics strategies specifically. How might you apply group dynamics going forward? • Take competency check
Social determinants of health	1. Ability to incorporate socio-ecological model for physical activity promotion in your county 2. Special population considerations (29, 30) 3. Knowledge of cultural, social, behavioral, and environmental factors that influence physical activity behaviors (29)	• Responses to quiz items—what surprised you? • Populations you've worked with—what environmental barriers did they face?	• Do a walking audit of your own neighborhood or nearby location • Email a picture or scan of your walking audit to your team prior to the next session • Take competency check
Policy, systems, and environmental approaches	1. Describe policy, systems, and environmental (PSE) changes to increase physical activity 2. List potential partners for physical activity PSE projects 3. Plan physical activity PSE projects that complement individual-level physical activity interventions (29)	• Email a picture or scan of your walking audit to your team prior to this session	• If you are not part of a local coalition addressing PA promotion, search online for a potential coalition to partner with for PA promotion and PSE • If you are already part of a coalition, • Write down how you would introduce PSE to your group • Share how you're already doing PSE work with this group • Take competency check
Partnerships for physical activity promotion	1. Educate, collaborate, and engage with external partners from a variety of disciplines to promote physical activity at multiple settings and in a variety of populations (29, 30) 2. Identify internal and external issues, such as changes and trends in financing, regulation, legislation, and policies that may impact delivery of public health physical activity services (29, 30)	• No break out this week	• Not on a coalition? How would you start one? Who would you invite to the table? • On a coalition? How did you show extension's PA efforts and knowledge? • Take competency check
Planning and evaluating PA programming	1. Use of framework or model to plan and evaluate physical activity interventions (29) 2. Knowledge of the physical activity readiness questionnaire (PAR-Q) 3. Knowledge of design, implementation, and evaluation of physical activity interventions to address chronic condition 4. Skill to analyze and interpret physical activity quantitative and qualitative data to validate conclusions (30) 5. Ability to produce and evaluation report and disseminate findings to stakeholders and decision makers (29)	• Why frameworks are important to guide your planning and evaluation • Overview of RE-AIM • RE-AIM for extension (with health equity considerations) • Physical activity readiness questionnaire PLUS • IRB approval (human subjects determination, research, program evaluation), and ethics • Use of mixed methods • Impact statement confusion	• Start with breakout this week: what are some challenges you face with evaluation?
Ready to set the PACE!	1. Audit and feedback on competency checks	• Recap • Feedback on competency checks—what you all got right/where we need to check in • What now? Strategies for existing programs: annual check in, deimplementation, scale out	• What is one thing you learned from PACE that you will incorporate in your work? What challenges remain? What should be included in PACE round 2?

*PACE, physical activity in Cooperative Extension; PAGA, physical activity guidelines for Americans; PA, physical activity; PSE, policy, systems, and environmental approaches; PAR-Q, physical activity readiness questionnaire; RE-AIM, reach, effectiveness, adoption, implementation, and maintenance model*.

Between the weekly synchronous sessions, support emails were sent to the entire participant pool that included a brief summary of the information covered during the synchronous session, as well as the homework assignments to complete for the asynchronous portion of the week. The recording of the week's session was not included in the weekly email, but was sent out to individual participants if requested.

### Measures

#### Reach and Representativeness

Participants were asked to report their sex, ethnicity, current job role, state of residence, and how long they had worked for extension. While agents were the target of the program, other extension personnel were not excluded; therefore, we have no denominator for the overall reach of the program. Outcome measures included pre- and post-program surveys that were completed online prior to and directly after the program, respectively. The post-program survey also included space for participant feedback. Attendance for all sessions was also assessed as a measure of ongoing reach. All surveys and post-session homework assignments were collected via Qualtrics (Qualtrics.com, Provo, UT).

#### Effectiveness

The Ext-PAPH knowledge, skills, and abilities (KSAs) used in this program were modified from the Essentials for Public Health Physical Activity Practitioners core competencies (29) and the Modified Version of the Core Competencies for Public Health Professionals (30). The National Physical Activity Society created the core competencies for public health physical activity practitioners in conjunction with the Centers for Disease Control and Prevention (CDC) and the American College of Sports Medicine (ACSM) (29). These three entities produced the knowledge, skills, and programming for the physical activity in public health specialist certification (29). Because extension professionals are not broadly public health specialists, but rather work within the directives of extension, competencies not covered in the specialist certification developed by the CDC and ACSM (social determinants of health, extension's role in physical activity promotion) were added to the modified core competencies for public health professionals. Eight total competency sections were included in the pre- and post-survey: (1) physical activity and public health, (2) extension's role in physical activity promotion, (3) selecting and adapting evidence based physical activity programs, (4) behavior change, (5) social determinants of health, (6) policy, systems, and environmental approaches, (7) partnerships, and (8) planning and evaluating. The competencies included a variety of questions on items such as extension's role in physical activity promotion (i.e., what extension personnel can promote, activities, and programs they can deliver); partnerships (i.e., building coalitions); and physical activity and public health (i.e., what are the physical activity guidelines and how can public health workers promote physical activity). Each of these eight sections had 2–6 competencies, with those competencies on a four-point Likert scale ranging from “none” (I am unaware or have very little knowledge of the skill) to “proficient” (I am very comfortable, am an expert, or could teach this skill to others). The max scores for each section ranged from 8 to 24, with a total possible Ext-PAPH score of 136. Neither the Essentials for Public Health Physical Activity Practitioners core competencies (29) nor the Modified Version of the Core Competencies for Public Health Professionals (30) have undergone validity and reliability testing, but were the closest measures of Ext-PAPH available in the literature (see Limitations).

#### Secondary Outcomes

##### Flourishing

To measure pre- and post-training flourishing, the VanderWeele secure flourish index (SFI) was employed. The SFI is broken into six domains (24): happiness and life satisfaction, mental and physical health, meaning and purpose, character and virtue, close social relationships, and financial and material stability (24). The last domain (financial and material stability) was included as an indicator of ability to sustain flourishing (23). The SFI has been validated in a workplace setting (23). Each domain is on a 0–10 scale and has two questions. Anchors were based on item language; examples included “not satisfied at all” to “completely satisfied,” “not true of me” to “completely true of me.” Higher levels of flourishing are shown through higher scores.

##### Physical Activity

The Godin Leisure-Time Exercise Questionnaire (31), a brief four-item questionnaire with items on mild, moderate, and strenuous activity as well as frequency of activity, was used as it has been utilized with a variety of different populations and shown to be valid (32, 33).

### Analysis

Descriptive and inferential statistics for all data were calculated using SPSS software (SPSS Version 26, IBM SPSS Statistics, Chicago, IL, 2020). To compare pre- to post-competency scores the non-parametric Wilcoxon signed ranks tests were used to note areas of significant improvement following the training. Flourishing scores pre- and post-PACE were compared using *t*-tests. Physical activity was categorized into meeting guidelines or not meeting guidelines based on methods proposed and validated by Amireault and Godin (32). Pearson chi square tests were used to determine changes in meeting guidelines. Data were analyzed via present-at-follow-up rather than intent-to-treat. Open-ended question responses were examined for feedback of the program.

## Results

### Reach

#### Reach Indicator 1. Total Number

One hundred thirty extension employees (including administrators, program leaders, and specialists) registered to attend PACE, 65 from Arkansas and 65 from Virginia.

#### Reach Indicator 2. Reach Proportions

Out of 190 eligible agents (128 from Virginia and 62 from Arkansas), 79 (41.6%) registered to attend PACE. Of all the employees who registered, 15 registrants (11.5%) registered late and then never attended a PACE session, with an additional 16 who registered on time but never attended a session. The majority (56.9%) of initial registrants attended all the sessions, and 64 (49.2%) completed the certificate.

#### Reach Indicator 3. Ongoing Reach

**Figure 1:**
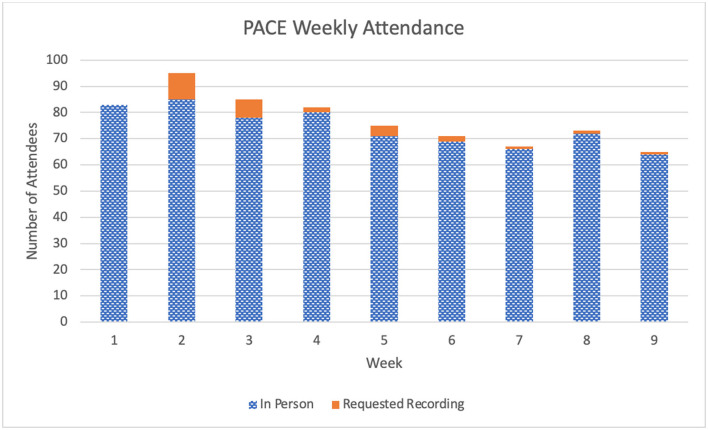
Weekly participant attendance for PACE.

Live attendance for each session was 77 (±6.0) participants, with 74 attending 100% of the sessions either live or via recording. Participants had the opportunity to request the recording of the session, with the most requested session recording being week 2 (Figure 1). Sixty-four participants (49.2%) completed the full micro-credentialing program, including the post-survey, and received their completion certificate.

#### Reach Indicator 4. Representativeness

The participants were 97% female, white (81%) or black (15%), and represented a variety of roles within extension; of whom, 61% were agents (i.e., the intended target audience). Demographics for extension as a system are unavailable. Completers of the program had been with extension for an average 8.57 years (±7.95 years). Those who registered late and then never attended a session had the most years with extension at an average of 10.53 years (±7.86 years).

### Effectiveness

The Wilcoxon signed-rank test indicated that after participating in PACE, the median scores had a significant, positive change for all competencies except extension's role in promoting physical activity. The results were as follows: physical activity and public health (Z = −6.411, *p* < 0.001); extensions role in physical activity promotion (*Z* = −6.040, *p* < 0.001); selecting and adapting evidence based physical activity programs (*Z* = −6.261, *p* < 0.001; behavior change theories and strategies (*Z* = −6.261, *p* < 0.001); social determinants of health (*Z* = −6.353, *p* < 0.001); policy, systems, and environmental approaches (*Z* = −6.202, *p* < 0.001); partnerships (*Z* = −6.152, *p* < 0.001); planning and evaluation (*Z* = −6.280, *p* < 0.001). Notably, seven and four participants decreased competencies for “extension's role in physical activity” and “partnerships,” respectively. Flourishing increased from 92.59 to 94.78, but not significantly, *p* < 0.09. Physical activity levels significantly increased (*p* < 0.05).

Open ended responses to survey questions were included to capture quality improvement/participant perspectives and did not undergo rigorous qualitative analysis. However, it is notable that many participants wanted to share personal limitations (physical or simply “new to this”) as well as excitement for physical activity and the PACE training. Feedback from participants while the training was ongoing included liking the interactive physical activity breaks, engaging with their small group members, and wanting even more interaction in the form of Zoom polls.

## Discussion

Public health workers can implement evidence-based programming, spread knowledge about physical activity guidelines to the public, and lead experiential learning opportunities (3, 15). Competency-based trainings have been shown to increase desire of participants to translate knowledge to others (13, 15, 22). A micro-credential provides employers with evidence that specific individuals within the workforce have specified training (25). Therefore, extension set the PACE through a competency-based physical activity training. Physical Activity in Cooperative Extension was shown to be effective in increasing Ext-PAPH competency scores, and had participants enthusiastic about what they learned. This program was successful in retaining participants who attended at least one session [65% in PACE when compared to 67% in other PAPH trainings (13)].

Regarding participant representativeness of typical extension demographics, it is difficult to know the true demographic profile of extension as a whole, as many times data are not made available at the state level or in the reporting of different studies. The majority of the program participants were white females. This is a similar demographic profile to other studies that have focused on agents who deliver health-based programming (11, 34, 35). However, 4-H, national nutrition programs, and family and consumer sciences (FCS), which are the typical areas within extension that deliver health-based programming, are only a few avenues for physical activity promotion within extension. Extension agricultural agents can also engage in these efforts, and a larger proportion of agriculture agents are male (compared to FCS and 4-H) (36). This iteration of PACE focused primarily on reaching agents delivering health-based programming, but future variations of PACE will strive to reach other agents as well (10). Representativeness data for comparison on participants' position (i.e., agent, administrator) within extension is also difficult to fully determine, as many studies do not report data on participants' position, duration in extension, and background/training (12, 37). Taken together, while this research brief aimed to compare PACE participants to non-participants within extension, the lack of these data at the state or national level makes this challenging. Simultaneously, the predominantly white and female demographic profile of the PACE participants limits generalizability. Future PACE efforts would benefit from a wider representation of program areas, sex, and race.

Ongoing reach can be operationalized as how much of the intervention was received (26). In this research brief, ongoing reach was operationalized within the program based on the denominators influenced by the diffusion of innovation theory (38). For example, approximately 65% of those who chose to come to at least one session attended all the sessions, finished the program, and earned their completion certificate. For participants who registered on time, retention was >50%. However, and despite the request to re-open registration, none of the individuals who registered late attended sessions (i.e., 0% ongoing reach). This suggests that those who learn of an intervention and register (almost immediately) were early adopters and more likely to complete the program.

Initially, 9 weeks of sessions and homework were proposed to cover the content and improve physical activity and flourishing within agents. Improving physical activity and flourishing of extension personnel was a goal of PACE based on research that increased physical activity of agents increases likelihood that those agents will lead physical activity programming (11). Physical health is also a part of flourishing, which includes life and job satisfaction (23). While physical activity both increased significantly after PACE, there was not sufficient time to fully detail other aspects of flourishing within extension, such as mindfulness and self-care.

Extension agents who are more active have been shown to be more open to starting physical activity programs within their state (11). To begin physical activity programming, agents must first be aware of the PAG, as well as extension's role in physical activity promotion. Based on the changes in Ext-PAPH competencies, the greatest number of participants improved in knowledge of PAG competency section vs. the change in the seven other competency areas (see Table 2). Learning more about PAG, the move your way campaign, and evidenced-based physical activity through a competency-based intervention may lead to increased delivery of community interventions (14, 15, 22) for physical activity and an increased proportion of Americans meeting the PAG.

**Table 2:** PACE participant pre- and post-change scores for the extension-based physical activity and public health competencies (*n* = 64).

	**Pre-training *M* (*SD*)**	**Post-training *M* (*SD*)**	**Change *M* (*SD*)**
	**(out of 4)**	**(out of 4)**	
Physical activity and public health	2.38(±0.58)	3.26(±0.38)	0.77(±0.56)^***^
Extension's role in physical activity promotion	2.25(±0.75)	3.23(±0.44)	0.85(±0.77)^***^
Selecting and adapting evidence based physical activity programs	2.14(±0.75)	3.07(±0.48)	0.94(±0.76)^***^
Behavior change theories and strategies	2.29(±0.68)	3.31(±0.49)	1.00(±0.72)^***^
Social determinants of health	2.19(±0.69)	3.21(±0.53)	0.91(±0.67)^***^
Policy, systems, and environmental approaches	2.22(±0.85)	3.32(±0.50)	1.10(±0.80)^***^
Partnerships	2.18(±0.82)	3.22(±0.51)	1.02(±0.76)^***^
Planning and evaluating	2.02(±0.75)	3.04(±0.48)	1.01(±0.75)^***^

*** *p < 0.001 via Wilcoxon signed ranks*.

While Ext-PAPH competency scores improved significantly overall from pre- to post-program, seven individuals showed decrease competency in the extension's role in physical activity section. This is worrisome in that the design of the program was meant to increase competency in physical activity and physical activity programs for extension personnel. This decline may be, in part, due to potential disagreement or initial misunderstandings of extension's role (e.g., participants who believe extension agents should serve as fitness instructors rather than deliver behavior change interventions). Other competency sections showed a few participants who reported less competency post-program, but overall participants gained knowledge.

Lack of peer-to-peer interaction has previously been cited as the roadblock for many participants in asynchronous trainings (16). By both using and teaching about group dynamics principles in a synchronous training through mechanisms such as breakout rooms on Zoom, individuals were able have peer-to-peer interaction and experience group dynamics in action. Through emails and answers to open ended questions on the post-survey, participants expressed that they enjoyed these aspects of the program and planned to incorporate them in their own programming moving forward. Participants also expressed a high desire to relate what they learned to their own program participants, which is in keeping with findings that competency based learning increases knowledge translation to others (15).

There were a number of further limitations to this work. The competencies used were the best fit for the program in their focus on public health and public activity. However, neither set of competencies that the Ext-PAPH competencies were drawn from has been tested for validity or reliability. Additionally, Godin is not a very sensitive measure, and may not be able to fully model the changes in physical activity that participant's experienced. Finally, there is no national repository of extension personnel demographics in order to formally compare PACE participants demographics to the extension personnel states or national demographics. This might lead to misinterpretation of the representativeness of the program participants.

Considering the promise of the program against the study limitations, a number of future directions are underway. Once the extension workforce has the basic information on physical activity programming, they will be better able to deliver evidence-based programs that already exist (12). PACE or Physical Activity in Cooperative Extension covered the physical activity in public health basics, with a goal of increased knowledge and competency of extension public health workers in. More modules to promote physical activity and flourishing via mindfulness and self-care have been developed and piloted. Validation of both Ext-PAPH and subsequent modules will be feasible once a larger sample is obtained. Once the competencies and format have been further studied (including in iterations outside the COVID-19 pandemic), there is potential for scaling out the program, using the same structure and core components to cover both other topics and other systems.

## Implications for Research and Practice

Physical activity is an important part of health and can be effectively promoted by public health workers including extension personnel. Competency-based trainings can improve dissemination of knowledge and evidence-based programming to the public, and PACE is a competency-based training that has been shown to significantly improve competency scores across the physical activity core competency essentials. These data support that public health workers in other settings will also benefit from competency-based trainings on physical activity in public health. The PACE program has engaged extension personnel and exhibited a high retention rate, leading to more extension personnel having confidence and skill in evidence-based physical activity programming.

Future iterations of PACE will expand to include more state extension systems and other public health workers, and monitor the long terms effects of PACE to see if participants incorporate more evidence-based physical activity programming. This could be evaluated based on number of physical activity programs delivered by PACE participants, or in a more general manner by reviewing reported physical activity support through grants obtained or partnerships formed to promote physical activity. Based on feedback from PACE participants, future efforts on recruitment and retention will focus on detailing the time commitment involved. Work is needed on the frequency and duration (i.e., dose) that fits within participants' work schedule while actually changing behavior, and also evaluating what additional training strategies, such as one-on-one instruction or further group dynamics-based trainings, are necessary to ensure increased competencies for all participants across all competency categories. The mechanism of effect, and the most effective group dynamics strategies—e.g., the rich discussion possible in small group break outs (interaction and communication) or establishing a team name (team distinctiveness) —should be explored.

## Data Availability Statement

The raw data supporting the conclusions of this article will be made available by the authors, without undue reservation.

## Author Contributions

All authors were significantly involved in the creation of the research questions and implementation of this research. AD analyzed the data and completed the manuscript. BD and LB assisted with editing and feedback. SH assisted with data analysis and manuscript editing.

## Funding

This work was supported by the Virginia Tech Open Access Subvention Fund.

## Conflict of Interest

The authors declare that the research was conducted in the absence of any commercial or financial relationships that could be construed as a potential conflict of interest.

## Publisher's Note

All claims expressed in this article are solely those of the authors and do not necessarily represent those of their affiliated organizations, or those of the publisher, the editors and the reviewers. Any product that may be evaluated in this article, or claim that may be made by its manufacturer, is not guaranteed or endorsed by the publisher.
